# A review of experimental task design in psychophysical eye tracking research

**DOI:** 10.3389/fnhum.2023.1112769

**Published:** 2023-08-17

**Authors:** Diego da Silva Lima, Dora Fix Ventura

**Affiliations:** Laboratory of Clinical Visual Psychophysics and Electrophysiology, University of São Paulo, São Paulo, Brazil

**Keywords:** eye tracking, visual psychophysics, preferential looking, perception, vision

## Abstract

While eye tracking is a technique commonly used in the experimental study of higher-level perceptual processes such as visual search, working memory, reading, and scene exploration, its use for the quantification of basic visual functions (visual acuity, contrast sensitivity, color vision, motion detection) is less explored. The use of eye movement features as dependent variables in a psychophysical investigation can serve multiple roles. They can be central in studies with neurological patients or infants that cannot comply with verbal instructions, understand task demands, and/or emit manual responses. The technique may also serve a complementary role, determining the conditions under which a manual or verbal response is given, such as stimulus position in the visual field, or it can afford the analysis of new dependent variables, such as the time interval between oculomotor and manual responses. Our objective is to review the literature that applied the eye tracking technique to psychophysical problems. The two questions our review raises are: can eye movements (reflex or voluntary) be an objective index of stimulus detection in psychophysical tasks? If so, under what conditions, and how does it compare with traditional paradigms requiring manual responses? Our (non-systematic) methodological review selected studies that used video-oculography as the technique of choice and had a basic visual function as their primary object of investigation. Studies satisfying those criteria were then categorized into four broad classes reflecting their main research interest: (1) stimulus detection and threshold estimation, (2) the effects of stimulus properties on fixational eye movements, (3) the effects of eye movements on perception, and (4) visual field assessment. The reviewed studies support the idea that eye tracking is a valuable technique for the study of basic perceptual processes. We discuss methodological characteristics within each of the proposed classification area, with the objective of informing future task design.

## 1. Introduction

Traditional psychophysical tasks require participant responses (such as key presses) that map unambiguously to a discrete or continuous variable. When the participant has his gaze monitored by an eye tracker, however, there are multiple ways to relate the measured gaze coordinates to stimulus detection. The continuous eye position signal must first be partitioned and labeled according to eye movement features (fixations, eye blinks, saccades, or smooth pursuit segments). Those features must then be related to the psychophysical variable of interest, such as whether a stimulus was detected, or whether the subject was able to discriminate some property of interest via a psychometric function. Due to the methodological questions that must be addressed during task design, we considered that the use of the eye tracker for psychophysical tasks merited a review article. While there are previous review works dedicated to the subject of perception during eye movements (Schütz et al., 2011; Binda and Morrone, 2018), we to develop a methodology-focused discussion spanning a broader set of research traditions.

In most research areas, mechanical and electric eye recording techniques have been replaced by video-based eye tracking (video-oculography) (Carter and Luke, 2020). The technique was first developed in the 1970s (Cornsweet and Crane, 1973; Merchant et al., 1974; Clark, 1975), and today it is part of a series of commercial systems with offerings at multiple points in the tradeoff between system cost and data quality. The last 10 years also saw novel custom-built and open-source eye tracking systems (Mantiuk et al., 2012; Hosp et al., 2020; Ivanchenko et al., 2021) bringing the promise of further reducing the cost to set-up gaze recording systems. In video-oculography, a 2D signal related to the displacement of the pupil center with respect to a corneal reflection (glint) is used. To map the measured displacement to a gaze position in the display plane, a calibration procedure is usually employed. The algorithm adjusts the gaze mapping function to the display dimensions, participant distance to display and the participant’s anatomical characteristics. Calibration procedures typically require the participant to fixate between 5 and 9 points on the screen while their eye position is measured. Calibration approaches based on geometrical modeling of the acquisition setup have been developed to minimize the requirements for calibration time or dispense with calibration altogether (Villanueva et al., 2009; Hathibelagal et al., 2015).

Eye tracking systems have four fundamental parameters that impact upon data quality (Duchowski, 2017; Gibaldi et al., 2017): temporal resolution (number of eye position samples measured per second), spatial resolution or precision (the non-systematic spatial error when reporting the pupil position), spatial accuracy (the systematic difference between true and reported pupil position), and operating distance (the range of distances the subject can be positioned relative to the camera). The temporal and spatial resolution impose limitations on what kind of eye movements can be studied: studies using basic point-of-regard measures such as relative fixation time on regions of interest can be evaluated with low-framerate systems (between 30 and 60 Hz). Fixational eye movements, however, due to their reduced amplitude, require higher-resolution trackers (typically 1,000 Hz temporal sampling rate and lower), and might require participant physical restraint using a chin rest.

Eye tracking has been central in several lines of basic perceptual research relating eye movements to perception. The now classical book by Yarbus (1967), for example, dedicates 4 of its 7 chapters to the relationship between stimulus properties and eye movements. Some of the effects described in this seminal work evolved into full research areas during the decades of 1960–1990. Examples of classical effects that have been described with the use of eye tracking techniques include the vanishing of visual contours after 1–3 s of stabilization of retinal projections (empty field effects), the importance of eye drifts and microsaccades for the integrity of perceptions during fixations, the role of blinks and saccades for perception and the prevalence of stimulus content over low-level visual features to predict preferred fixation location in complex scenes (Riggs et al., 1953; Yarbus, 1967).

While traditionally the technique has been used to study the relationship between the oculomotor system and perception, multiple other demands have driven its use in psychophysical studies. The measurement of eye movements is more appropriate than manual responses for certain participant populations such as children or neurological patients that might not be able to cooperate in tasks requiring manual responses. Even when evaluating more cooperative populations, the high information rate afforded by the technique (that gives many samples per second rather than one discrete response every few seconds) has the potential to decrease session times and participant fatigue. Eye tracking also has the potential to improve the reliability and ergonomics of visual field examinations, since tasks can be designed to adaptively change the spatial position of stimuli based on the current gaze position, instead of requiring the participant to maintain fixations at the same point for long periods of time.

## 2. Overview and study inclusion criteria

The present review article aims to present the current methodology in eye tracking technique applied to psychophysics. The studies included were found by conducting searches over the databases PsycNET (American Psychological Association) and Web of Science (Elsevier), with the Cartesian product of the terms “eye tracking” or “video-oculography” with the terms “acuity,” “contrast sensitivity,” “psychophysics,” “spatial frequency,” “motion,” “temporal frequency” or “visual field.” The search was performed over the title, abstract and/or keyword fields. Since the review focus was on basic visual functions, studies aimed at investigating higher cognitive functions (such as attention or memory) or socio-emotional aspects were not included. Works that were referenced in any returned results from the examined databases that also happen to fulfill the inclusion criteria were considered for inclusion.

To further limit the scope of the present discussion, studies using eye tracking techniques that do not involve video-oculography (such as electro-oculography) were excluded, as were studies that used video-oculography but had pupil diameter (pupillometry) as their fundamental dependent measure. Only studies published in English as research articles were considered for inclusion.

In this review, we attempted to group studies based on their methodological similarity, as means of making common research themes and techniques more evident. The review focuses mostly on work done in the last decade (2010–2022), due to the relatively high volume of publication in the general eye tracking literature during this period (Carter and Luke, 2020), which also happened to contain most studies fulfilling the proposed inclusion criteria.

A total of 29 studies satisfying those inclusion criteria are discussed in the present review. We divided our review into four main parts. The first part presents studies that use different eye movement features as measurements of spatial vision thresholds (15 studies). In the second section, we review a few studies dedicated to study the effect of stimulus properties on fixational eye movements (3 studies). In the third section, we review modern literature on the research area of perception during eye movements (4 studies). In the fourth section, we review the literature in the area of visual field research (7 studies). Studies were presented in chronological order of publication within each of the subsections of those defined categories. The works considered for review are summarized at Supplementary Table 1.

## 3. Eye movements as a measure of stimulus detection

Gaze is directed toward regions of the visual field with salient features, a process that is extensively studied in visual search tasks (Wolfe, 1994; Loschky and McConkie, 2002) and was demonstrated in babies by Fantz in the 1950s (Fantz, 1958). Low-level visual features such as spatial frequency and contrast define a saliency value of a given region in the visual field, and gaze tends to be directed toward the position in the visual field with the most salient feature in a “pop-out” or “winner takes all” effect (Itti and Koch, 2000; Schütz et al., 2011). The occurrence of saccades near stimulus onset and the prolonged fixation on salient stimuli can then be used as a measure of detection for observers in psychophysical tasks. The two next sections are dedicated to reviewing studies that used saccades and fixations as indices of stimulus detection under different conditions. In the first subsection, we review studies that measured saccades immediately after stimulus onset and employed explicit instructions for fixations (reactive saccades). In the second subsection, we review studies that simply tried to characterize participant visual exploratory behavior, in situations in which participants were under generic instructions or under no instructions as to where they should fixate (preferential looking paradigm).

### 3.1. Reactive saccade measures of stimulus detection

Saccades are fast conjugate eye movements that are either elicited due to sudden or highly salient stimuli in the periphery of the visual field or are under voluntary control during the execution of visual tasks or scene exploration (Leigh and Kennard, 2004; Gremmler and Lappe, 2017). While the term reflexive saccade is common in the literature to designate saccades of the first kind, we will use the terminology of Gremmler and Lappe (2017) (reactive saccades) to designate such movements, to avoid confusion with eye movements under exclusive control of reflexive neural circuits such as the optokinetic nystagmus (OKN) response. Saccades can be extracted from the raw gaze signal either by identifying regions with high velocity (Nyström and Holmqvist, 2010), or by exclusion, by first identifying regions with low dispersion (fixation intervals), then labeling regions failing to fulfill those criteria as saccades (Blignaut, 2009). By arranging stimuli spatially into a few fixed positions, participants may use saccades to respond in an N-alternative forced choice (AFC) fashion in a way analogous to button press responses. Alternatively, by presenting stimuli anywhere in a circular arrangement, the angular displacement of the saccade target and the stimulus position can be used as a measure of detection.

Kilpeläinen et al. (2013), for example, showed that mean luminance changes have an attenuating effect on fixations toward high-contrast stimuli. Eight adult observers with normal vision participated in a task with instructions to fixate into one of a pair of sine wave gratings presented simultaneously at 7° eccentricity from the fixation point (separated by a 30° polar angle) superimposed on a circle with a radius of 10° eccentricity. The circle was divided into 12 sectors. Each sector had one of four possible luminance values. Those stimuli were presented on a 22-inch liquid crystal display (LCD) display, and eye movements were recorded with an EyeLink 1000 system at 1,000 Hz. If target gratings were presented on sectors with steady (low or high) luminance, saccades landed on the midpoint between targets according to a “center of gravity” effect. However, if the mean luminance of one of the targets changed immediately before the target presentation (step target), saccades deviated away from the midpoint between targets, with a tendency to deviate toward the target with steady background luminance, an effect that is related to the decreased contrast (and therefore visual saliency) of the step target relative to the stable target.

Manual (key press) and eye tracking responses were compared in a study by Zhuang et al. (2021). They were collected in a 5AFC contrast sensitivity (CS) task in which subjects had to inform the grating position among one of the four cardinal directions or display center. The subjects were 27 healthy young adults with normal viewing conditions and lens-induced blur. Monocular gaze position was measured with an EyeLink 1000 eye tracker. Subjects were instructed to look toward the stimulus when they detected it, or at a central blank area when they did not detect it. The task used a 3-down 1-up staircase, (varying the stimulus contrast by 10% at each step). Detections were scored whenever participants fixated within a circular area around the stimulus for at least 1 s during a maximum of 6 s of presentation time. The authors found a good correlation between eye movement responses and manual responses (*r* = 0.966; *p* < 0.001) with a coefficient of repeatability of 0.377 log CS across different days. A small but statistically significant difference in CS scores was reported for each spatial frequency, however, with lower thresholds for manual responses than for saccadic responses. The authors attributed this difference to visual fatigue and pointed out that refinement in the choice of adaptive psychophysical procedure might decrease testing time to minimize this source of variability.

In order to investigate peripheral contrast sensitivity, two conditions were compared by Essig et al. (2022): before reporting stimulus orientation (45, 90, 135, and 180°), participants were asked to either perform a saccade toward the stimulus after its offset or to maintain fixation on a central point. A total of 12 adults with normal vision participated in the task. Eye movements were recorded with EyeLink 1000 Plu at a sampling rate of 1,000 Hz. An adaptive presentation scheme was used. The CS measured with saccades only, button presses after saccades, and button presses without saccades were all correlated (*r* ≥ 0.79). The motivation for this procedure was to have a measure of peripheral contrast sensitivity, since previous studies had focused on central contrast sensitivity (via OKN or fixational eye movement measurements).

For adult participants that understand verbal instructions, saccadic movements can be used as voluntary responses to select one from a small set of alternatives. The studies that used eye tracking in a forced-choice design (Zhuang et al., 2021; Essig et al., 2022) were found to be mostly comparable with manual responses, suggesting the use of the technique without many changes from the traditional design in situations where manual responses are not possible, such as the evaluation of neurological patients that can comply with verbal instructions. When faced with conflicting peripheral stimuli, a “center of gravity” effect may be observed. This effect is considered to result from the integration of conflicting stimulation of different regions in the superior colliculus retinotopic map that resolves to the vector average of the two stimulated regions (Kilpeläinen et al., 2013). Such an effect of spatial conflict cannot be observed with button press responses and may be explored to answer other research questions.

### 3.2. Fixation-based based measures of stimulus detection (preferential looking)

The preferential looking technique relies on the spontaneous tendency of observers (including infants and non-human primates) to fixate on more complex portions of a visual stimulus (for example, to fixate first and/or for a longer time into a grating pattern rather than a homogeneous background with the same luminance). This natural tendency may be used for psychophysical testing if some property of interest, such as the pattern contrast or the spatial frequency, is varied in a fixed or adaptive procedure up to the point where this preference disappears when the stimulus is below the threshold (Fantz, 1958; Teller, 1979). The technique has been used in several commercially available tests that have been validated for diagnostic use, such as the Teller Acuity Cards (Teller et al., 1986; Salomao and Ventura, 1995) and the Hiding Heidi contrast sensitivity test (Chen and Mohamed, 2003). Traditionally, the technique relies on trained examiners that observe the participant and make a forced-choice or qualitative judgment about aspects of the participant’s non-verbal behavior that suggest the stimulus was detected (such as head orientation and changes in gaze direction toward the stimulus). Naturally, several studies examined the possibility of using eye tracking to automate this process. While the studies described here are similar to the studies in the previous section in that voluntary eye movements are used as the detection criteria, their dependent measure is based on the observer’s spontaneous visual exploratory behavior as a measure of stimulus detection, rather than on explicit instructions to fixate the stimuli. In eye tracking preferential looking studies, instead of extracting the target of the first saccade after stimulus onset as the dependent variable, relative fixation time on the region of interest throughout the presentation trial is usually the variable of interest.

In order to use a preferential looking task to estimate visual acuity (VA), Sturm et al. (2011) tested nine adults presented with four luminance patches on monitor [21-inch cathode ray tube (CRT) display], three of which were homogeneous luminance and the fourth was a horizontal square wave grating pattern varying in spatial frequency in 14 steps. Participants were instructed to look toward the monitor, but no specific instruction to look at the target was given. Eye recordings were made with a custom eye tracking system that sampled at 30 Hz, used two or more corneal reflexes, and required a single calibration point. The authors used the relative fixation time (time fixating at the grating divided by time fixating at other patches) as their dependent measure. The detection of each grating was determined as the likelihood ratio of the empirical distribution functions calculated based on the psychophysical task (that exceeds a threshold for a fixed acceptable rate of false positives of 5%). The VA was found to be underestimated on average by 0.11 logMAR relative to conventional psychophysical thresholds (ranging between −0.73 and 0.37 logMAR).

Jones et al. (2014) built a protocol for measuring visual acuity (ACTIVE) and compared their results with a traditional clinical test for infant visual acuity, the Keeler Infant Acuity Cards (KIAC). The commercial Tobii TX120 system was used (sampling at 60 Hz). The authors developed a novel calibration procedure with infants. In this procedure, a default observer calibration model was applied at first (developed based on the spatial distribution of detectable stimuli on the screen), and the system adaptively corrected the gaze position by calculating systematic differences between the current gaze positions and the current state of the model. A LCD display of 30 inches was used to present the stimuli, and participants sat at a distance of 84 cm. Gabor patterns were presented against a gray background, and their position at each trial was calculated from a random angle in a circumference with a constant radius presentation of 8°, centered at the current infant point of fixation. As highlighted by the authors, the measured acuity values are at best a lower bound on the acuity value, since at stimulus onset, the participants detect it at a parafoveal region. The patterns varied in spatial frequency according to an adaptive up-2-down-1 staircase. The acuity values obtained in a sample of 30 children between 2 and 12 months of age agreed with the traditional test (86% of examinations differed in less than 0.3 logMAR) and with the conventional KIAC test norms (78% of values were within the normative limit interval).

A similar procedure to evaluate visual acuity is described by Hathibelagal et al. (2015), who tested 19 children with normal vision between 3 and 11 months of age. Stimuli were horizontal square wave gratings, presented at one of 4 locations of a 21-inch LCD display [in a stimulus arrangement similar to that of Sturm et al. (2011)]. Participants younger than 6 months old sat at 70 cm from the monitor, and older participants at 120 cm. In a custom-built system, the authors used a pair of Point Grey Grasshopper 20S4M-C cameras (30 Hz sampling rate), with a reported accuracy of 0.5 degrees and spatial resolution of 0.1 degrees. The two-camera setup is used to determine the optical axis of the participant using a geometrical model (based on the intersection of the two planes formed by camera-corneal-reflection-pupil at the two images), and does not require a calibration procedure. Decisions on whether participants fixated on the gratings were made based on real-time inspection of the gaze path and bar graphs that showed the proportion of time the participant fixated on the grating instead of the other three regions without the grating. The grating was considered seen if at least one of the following criteria were met: (1) the first fixation after stimulus onset was on the grating and the fixation persisted for at least 2 s; (2) The proportion of time spent fixating the grating was at least 75% of the stimulus presentation time. Acuity values agreeing across two evaluation sessions (differences smaller than 0.3 logMAR) were found in 89% of children, and the differences between their test result and the Teller Acuity Cards were less than 0.3 logMAR in 74% of cases at the first test and 58% of cases at the second test.

Another tool developed to assess visual acuity was described by Vrabič et al. (2021), who compared the Keeler Acuity Cards and Lea symbols with an eye tracking technique in 36 healthy children with ages between 5 months and 16 years old. Stimuli were presented on a 15.6-inch laptop display, in a 2AFC design with stimuli presented randomly at the left or right portion of the screen. A commercial Tobii Pro X3-120 system sampling at 120 Hz was used, with a 5-point procedure used for calibration. The ratio of the time spent fixating on the target stimulus to the time spent fixating on both left and right stimulus regions was used to determine stimulus detection, with two candidate thresholds for this ratio set at 60 and 75%. The exams agreed with an *r* = 0.53% (0.31–0.72 CI) The authors identified that in the subsample of children with low acuity (> 0.4 logMAR) the test overestimated the acuity relative to standard testing; and underestimated the acuity in children with high acuity (< 0.4 logMAR).

A similar study was conducted by Chang and Borchert (2021), who applied the eye tracking technique to assess visual acuity in a clinical population. Vertical square wave gratings against a homogeneous gray background were presented at either the left or the right side of a 24-inch display screen. Eye movements were recorded with an EyeLink 1000 Plus (sampling at 500 Hz and with spatial resolution smaller than 1°). A 3-point calibration procedure was used, and participants were instructed to just look at the screen during the display of the stimuli. Detection was established when the patient fixated on the pattern for at least half the presentation time of 2 s. Sixteen children with cortical visual blindness between 12 months and 12 years of age were tested. The authors compared the result of the Teller Acuity Cards with the eye tracking examination, having found a high correlation between the two techniques (*r* = −0.82) and high test-retest reliability (*r* = 0.96).

Another study with a clinical focus was the recent work by Esteban-Ibañez et al. (2022). The authors evaluated visual acuity and contrast sensitivity in children between 6 months and 7 years old through eye tracking. The authors named the procedure DIVE (Device for Integrated Visual Examination). The patients were examined using a small, 12-inch display with a custom eye tracker sampling at 60 Hz. Stimuli were vertical square-wave gratings presented at one of four circular patches that covered the central portion of the screen. Positive verbal?? feedback was given, contingent on fixations over the correct target. The eye tracking system was calibrated using a 9-point procedure. Two phases of the study are reported: in the first, 60 patients without visual problems were tested to establish normative test data. Visual acuity was successfully estimated in 57 patients, while 44 were tested successfully in contrast sensitivity. In the second phase, 74 patients were tested, divided into a group of 28 patients with normal development and 41 patients with clinical conditions, potentially related to visual function (31 were preterm; 5 had low gestational weight; 3 had congenital cataracts and 2 had congenital nystagmus). In this second study, 69 patients could complete the test. Trends between age and visual function were identified both for visual acuity and contrast sensitivity. The difference between the Lea grating test and the eye tracking test visual acuity test was 1.05 cpd (CI = −9.95 to 7.84 cpd). The authors noted that the differences between the two tests were smaller for children younger than one year old.

The works dedicated to establishing the equivalence between traditional preferential looking and eye tracking-based preferential looking show statistically significant correlations between results from the two techniques, but also small differences in measured thresholds (Sturm et al., 2011; Vrabič et al., 2021; Esteban-Ibañez et al., 2022). The eye tracking preferential looking tasks applied with infants and children do not require head stabilization or strict instructions to fixate at any given point in the screen, but rely instead on spontaneous visual exploratory behavior. While task administration is simple and imposes little demand on participants, studies must specify relevant criteria of stimulus detection, since the child does not receive any type of instruction. This can make it challenging to compare the results of different studies, since each study uses different criteria for stimulus detection, based on either heuristics or pilot data that are specific to each task.

### 3.3. Pursuit-based measures of stimulus detection

An advantage that the eye tracker affords, which has no parallel with manual or verbal responses in psychophysical testing, is the use of reflex and involuntary responses. Smooth pursuit is a conjugate eye movement with speed and trajectory that reproduces the dynamics of a continuously-moving stimulus. Unlike saccades, smooth pursuit movements cannot be initiated voluntarily, but must be initiated in the presence of a moving stimulus (although it can also start based on predicted stimulus motion before stimuli start moving) (Missal and Heinen, 2017). The movement persists up to the point where a tracked object disappears or stops [but the movement may persist for short periods of target occlusion (Missal and Heinen, 2017)]. Measures of pursuit movements have the advantage that they are at least in principle more robust against variables such as participant motivation and individual differences in visual attention due to their involuntary nature. Pursuit-based measures, in comparison with saccades and fixations, have the potential to be unambiguous indicators of stimulus detection due to the close relationship between stimulus and gaze trajectory (Schütz et al., 2011).

Ming et al. (2016) used eye tracking to investigate the effects of eye movements on the spatial and spatio-temporal contrast sensitivity function of patients with Parkinson’s disease (PD), given that this condition may cause problems of fixation stability and impaired pursuit movements, thus leading to the hypothesis that the perception of dynamic stimuli might be more impaired than the perception of constant stimuli. Patients with idiopathic mild to moderate Parkinson’s disease (*N* = 13) and age-matched controls (*N* = 12, mean age of 66.8 years, standard deviation of 6.8 years) viewed stimuli on an 18-inch CRT display while their eye movements were recorded with an EyeLink 1000 (at 1,000 Hz and spatial resolution of 0.01°). Static and horizontally-moving Gabor stimuli (that participants had to track with their eyes) were presented, and participants were asked to adjust the stimulus contrast on a continuous scale until the patterns were barely visible. The authors observed a motion gain phenomenon (increased CS in lower spatial frequencies for moving stimuli) in both controls and patients. Patients had spatial contrast sensitivity decreased relative to controls only at the middle spatial frequency ranges. PD patients showed 53% more microsaccades (defined as saccades with less than 0.5° in magnitude) than controls. The authors also demonstrated decreased spatial frequency thresholds and a spatial frequency peak shift (from 4 to 1 cy/deg) when using dynamic stimuli. Differences in CS functions between patients and controls were not significant for dynamic stimuli.

The main motivation of a newly-described methodology denominated by the authors “Curveball” (Mooney et al., 2018) was to decrease the time required to administer psychophysical tasks, with the aim to minimize low engagement resulting from long exposure to repeated stimuli near threshold, and also to be able to study cognitively impaired populations with brain injury but with preserved eye movement. The authors inspired their task on “continuous psychophysics,” (Bonnen et al., 2015) in which the participant performance is measured as a continuous function of time, instead of being calculated from a set of discrete choices. In their tasks, 35 adults with normal or corrected-to-normal vision followed a circular stimulus (narrow-band frozen noise patches of 12°) along an unpredictable circular path in a screen. A 27-inch LCD display was used to present the stimuli, and a Tobii 4C eye tracker was used for recording (at 60 Hz sampling rate). The CSFs calculated with the procedure were found to be similar to CSFs calculated with a traditional staircase procedure, and the task could be completed in approximately 5 min.

Mooney et al. (2020) also presented a novel procedure, named “Gradiate,” for the measurement of contrast sensitivity function (CSF). Sixty observers were presented with moving stimuli, with the same display and tracking system setup as the previous study. In this new procedure, the stimuli varied in contrast along their trajectory, but with differing starting spatial frequencies. As the target moved on the screen, an online algorithm continuously checked if all the last 8 gaze samples were close to the corresponding 8 points of the stimulus central position (within 0.4°). Each participant completed four repeats of six spatial frequency runs (0.25, 0.5, 1, 2, 4, and 8 cpd). The correlations of CSFs calculated with this and with traditional tasks were moderate (*r* = 0.681 ± 0.170).

Traditional contrast sensitivity procedures are time-consuming and require focused attention over long periods, thus not applicable to neurological patients (Mooney et al., 2020). Pursuit movements may not only decrease testing time (since a higher number stimulus values can be presented continuously over time) but might also decrease false-positive rates when compared with eye tracking studies that use voluntary eye movements (Mooney et al., 2020), since the eye movement dynamics of pursuits cannot be reproduced in the absence of a stimulus. Due to the dynamic nature of the stimuli used, CSFs measured with pursuit movements have been found to be different from CSFs measured with traditional tasks using static stimuli (Mooney et al., 2020), so applicable normative data still need to be established before more widespread use.

### 3.4. Reflex-based measures of stimulus detection

The optokinetic nystagmus (OKN) is an ocular reflex response to moving stimuli in the visual field. This involuntary response is composed of a slow phase in the direction of the moving stimulus and a quick phase in the opposite direction that repositions the eyeball in a resting state. The two phases alternate in a saw tooth pattern while the stimulus persists (Essig et al., 2021). Several recent studies explore its use to determine psychophysical thresholds, either by changing some property of the OKN-inducing stimulus or by changing some property of some stimulus superimposed on the fixation point used to inhibit an OKN response. Hyon et al. (2010) studied the possibility of using the OKN reflex to evaluate visual acuity in healthy adults. Participants were 83 adults with normal vision, 56 of which performed the test without refractive lenses, and 27 with lens fogging. Participants maintained their heads at a distance of 305 cm from a 127-in projection screen. High-contrast black and white stripes moving horizontally served as the OKN-inducing stimuli. In an induction condition, the stripes increased in spatial frequency up to the point where an OKN response could be measured. In a suppression condition, the central fixation dot increased in diameter up to the point where the OKN response could not be detected anymore. The linear correlation between visual acuity measurerments with the OKN and manual responses was statistically significant (the *R*^2^ between the OKN and manual responses was 0.566 for the induction method, and 0.832 for the suppression method).

The OKN reflex response was compared with a traditional psychophysical contrast sensitivity paradigm by Dakin and Turnbull (2016). Adult participants (*n* = 30) with normal or corrected-to-normal visual acuity were presented with a leftward or rightward drifting noise on a 22′ CRT display at 72 cm. They were asked to make subjective judgments of the direction of the stimuli, which were rectangular (34.5° × 26.2°) two-dimensional Gaussian noise patches blurred with an isotropic two-dimensional filter. The authors developed an algorithm to separate the tracking phase and the saccade phase of the OKN reflex based on the velocity of the eye position signal. A 9-point calibration procedure was used. The authors found a high (*r* = 0.95) correlation between the thresholds calculated by the OKN-based technique and the button-press 2AFC psychophysical task.

Schwob and Palmowski-Wolfe (2019) compared the optotype-based Freiburg acuity and contrast test (FrACT) with a novel test named “SpeedWheel” in the evaluation of 15 school-age children (6–12 years old) and 27 adults with refractive errors and/or amblyopia or associated visual conditions Participants had to discriminate the orientation of the optotype (4AFC for the “E” optotype and 8AFC for the Landolt-C optotype). Both tests were viewed on a 28-inch LED display at a distance of 1 meter. For the novel procedure, an OKN-inducing pattern of black-and-white vertical stripes moved horizontally at 10°/sec from left to right. A set of dark dots arranged in a cross pattern was then presented around the fixation point, with continuously increasing diameter. The objective of the task was to determine at which spatial frequency of this pattern the OKN response was inhibited. Inhibition of the response was determined by visual inspection with the help of mobile eye tracking glasses. The mean difference against the mean was −0.01 for the Landolt-C optotype and −0.15 for the E optotype. The correlation of Speed Wheel??? tests results with FrACT was high (*r* = 0.85 for Landolt-C, *r* = 0.81 for the E optotype, *p* < 0.001; although values were smaller for children with *r* = 0.69, *p* < 0.005 for the Landolt-C and *r* = 0.74, *p* < 0.003 for the E optotype).

OKN-measured contrast sensitivity was also studied by Essig et al. (2021) using a sine wave grating drifting over the horizontal plane (with a velocity of 2.3/deg/s-1 presented on a 1,920 × 1,200 px display at 0.252 pixel pitch). The participants (*N* = 15) had normal vision and were tested at 75 cm. The stimuli varied in spatial frequency between 0.7 and 6.5 cy/deg and contrast was changed from a low to a high value (between 0.03 and 66%). The presentation was interrupted as soon as an automated analysis of the gaze data showed the presence of an OKN presence. Eye movement recordings were made with an EyeLink 1000 Plus system with a 1,000 Hz sampling rate and a 9-point calibration procedure. The contrast sensitivity functions calculated from the procedure followed the expected profile (parameters for a log-parabola function fit were estimated with *R*^2^ > 0.84), and the authors found that the application of defocus lens between +1.5 and +2.5D (used to simulate low-vision conditions) reliably decreased CS for high spatial frequencies.

The advantage of using the OKN reflex as a measure of stimulus detection is that subjects cannot voluntarily inhibit it (Howard et al., 1989). Its application, however, is limited to participants that can maintain fixation for a reasonable interval of time. The extraction of OKN patterns from raw gaze data is a well-studied problem for which a series of algorithms have been developed. Dakin and Turnbull (2016) describe in detail their solution based on the calculation of horizontal gaze velocity, extraction of the sign of this velocity signal depending on the direction of the stimuli, and classification of extracted regions as either a saccadic or tracking phase of the OKN response based on the magnitude of the velocity signal peak. Essig et al. (2021) use a modified version of a classical algorithm for microsaccade extraction (Engbert and Kliegl, 2003) to detect the saccadic phase of the OKN response. To detect the slow phase, the authors examined the segments before each saccade for a predominant velocity profile consistent with what would be expected from the stimulus direction. Once an algorithm is established to reliably separate segments with OKN response from segments that do not present OKN response, psychophysical thresholds can be established.

## 4. Effects of stimulus properties on fixational eye movements

While the eye is fixating on a stimulus, the oculomotor system produces a series of small-amplitude movements. Fixational eye movements are classified into three basic categories: drift, tremors, and microsaccades (Engbert and Kliegl, 2003). Drift movements are small amplitude movements with random direction, while tremors are small amplitude oscillatory movements superimposed on drift movements. Microsaccades are quick, binocular, directed movements with nearly linear trajectory occurring at a rate of 1–2 per second with amplitude between 1′ and 25′ (Engbert and Kliegl, 2003), whose occurrence corrects the natural eye drift during fixations (Denniss et al., 2018). Some studies have explored the possibility of using differences in fixational eye movements across different stimulus conditions for the objective quantification of visual functions.

Microsaccade inhibition has been shown to be a valid indicator of stimulus detection (Bonneh et al., 2015). Gabor patches flashed in intervals of 100 ms at 1 Hz were presented to 20 observers with normal vision and the relative frequency of microsaccades (relative to each participant’s baseline rate) across different spatial frequencies and contrast levels was measured. The microsaccade rate modulation function showed (1) anticipatory early inhibition prior to stimulus onset (due to the repeated nature of the stimulus), (2) stimulus-dependent inhibition that reaches a maximum at 150–200 ms after onset and (3) contrast-dependent and spatial frequency-dependent release from inhibition.

Scholes et al. (2015) studied how microsaccade signature may change as a function of stimulus contrast. Seven adults with normal or corrected-to-normal vision maintained fixation on a central white dot while Gabor patches with varying contrast and random phase were presented centrally. Participants positioned their heads on a chin rest while observing the stimuli on an 18-inch CRT monitor. Eye recordings were made with the EyeLink 1000 at 500 Hz. A 9-point calibration procedure was used. Subjects were tested on a passive condition (where they were asked just to maintain fixation while the stimuli were presented) and a 2AFC condition to which the orientation of the stimulus had to be reported using a keypress. The typical microsaccade rate signature in a baseline condition (in the absence of stimulus) is characterized by an initial inhibition phase, followed by an elevated rate during stimulus presentation. In the study, both the inhibition and rebound magnitudes of the rate signature were found to increase non-linearly with stimulus contrast. A support vector classifier trained with the microsaccade rate feature could adequately predict the participant’s psychophysical thresholds [root mean square (RMS) error = 0.072].

Denniss et al. (2018) had as the main objective using microsaccade inhibition to calculate contrast sensitivity thresholds. Patients with normal vision (*n* = 19), amblyopic (*n* = 10), or with cataract (*n* = 9) were tested on a 2AFC task (to discriminate between 45 or 135° Gabor stimulus orientation) and on passive viewing of a salient stimulus task, with microsaccades rates compared. An EyeLink 1000 system was used for data acquisition at 1,000 Hz, and a CRT display (with 1,024 × 768 resolution) was used to present stimuli. In a first psychophysical procedure, the stimulus contrast threshold for each trial was established via a 3-down 1-up staircase procedure. The calculated CSF for this first procedure was then used to determine the contrast levels of the eye tracking task, in which the stimuli were presented around a fixation point according to a method of constant stimuli at 7 different levels. For some of the trials, participants had to simply maintain fixation; while for other trials, they had to respond to the stimulus orientation as before. Correlations between the two methods were high (rho = 0.74, *p* < 0.001), with the drawback that the eye tracking method required 20 times more stimulus presentations than the psychophysical method. The authors identified high heterogeneity of the microsaccade rate signature, that could not be attributed to age, contrast sensitivity, or clinical condition.

The recording of fixational eye movements requires eye tracking systems with higher spatial resolution, since they are much smaller in magnitude than typical saccades. An algorithm for microsaccade extraction is described by Engbert and Kliegl (2003), based on a multiple of the standard deviation of local velocity segments as the velocity threshold, and the local velocity median as the value to be used for detection, with a binocular constraint that the temporal interval off saccades with two eye measurements should overlap. This algorithm was used by Bonneh et al. (2015) and Scholes et al. (2015).

## 5. Effects of eye movements on perception

The eye tracking technique plays a pivotal role in studying perception during eye movements, since timing the onset and offset of those movements relative to stimulus presentation, as well as their trajectory. Both fixed or moving stimuli with a moving eye generate spatiotemporal patterns on the retina that must be integrated across time and space. How this integration happens is an old research area, with some theoretical questions still in dispute. There is a rich literature on perception during eye movements. A complete review of the effects of pursuit eye movements on perception was done by Schütz et al. (2011). A review of perception during saccadic eye movements is provided by Binda and Morrone (2018). We present here just a few studies of this research area, to evaluate their implications for the current methodological discussion.

By relating the timing of eye movements to manual responses, processes such as perceptual stability can be studied. Schütz et al. (2007) investigated the effect of smooth pursuit suppression on the contrast sensitivity threshold. Participants were asked to fixate on a dot moving horizontally through the screen while a blurred low-contrast horizontal line with varying contrast was presented at a vertical offset (top or bottom of the screen), to which the subjects had to make a forced choice. Eight adults served as subjects. The experiments showed no effect of pursuit onset on the stimulus detection for the ramp stimulus movement; but showed a pronounced saccadic suppression effect for a step-ramp movement, that required a saccade at stimulus onset. The saccadic suppression effect (the inability of acquiring visual information during saccades) is a well-known effect that has been characterized as early as the 1890s in reading studies (Matin, 1974). The effect can be informally verified by trying to read a line with just two horizontally displaced fixation points, one at the beginning of the line and one at the end (readers are usually unable to report what was written). Thresholds for the detection of flashing stimuli were classically demonstrated to be higher during saccades than during fixations (Volkmann, 1962). There was disagreement on whether the visual stimulation is actively inhibited by the brain during saccades or simply a result of the eye not having sufficient time to integrate the visual information temporally during the saccade, or quick superposition of the retinal input at different points in time (temporal masking) (Castet and Masson, 2000; Gremmler and Lappe, 2017).

Castet and Masson (2000) studied intrasaccade motion perception by a moving grating (in the same direction as the saccade), examining how this motion perception differs at different relative velocities of the grating with respect to the peak saccade velocity. Stimuli were presented in a 21-inch display during different portions of the saccade interval. Eye movement was measured with a high-resolution infrared scleral-reflectance system (IRIS Skalar) at 500 Hz. In the moving condition, low spatial frequency gratings were presented in a horizontal motion of 300 or 360°/s in the same direction as the participant made a saccade between two fixation points. Three adult observers participated in the task, and they had to make a judgment about whether grating motion was perceived. Saccade velocity was manipulated by varying the horizontal distance between fixation targets, exploring the fact that larger saccades generate larger peak velocities. The probability of perceiving motion was found to be non-linearly related to the saccade peak velocity following an inverted-U shape, suggesting perception of motion during the saccade depended on the short period of high, constant acceleration in the middle of the saccade interval where the temporal frequency of the projection of the grating stimulus on the retina is most stable.

The saccade suppression effect was also studied by Gremmler and Lappe (2017). The authors used an EyeLink 1000 system sampling at 1,000 Hz to monitor the participant’s gaze and a 22-inch monitor to present stimuli. In their design, they included a voluntary saccade condition in which subjects were instructed to direct their gaze away from a central dot fixation point and toward a lateral dot only after some time was elapsed after the central dot disappeared, and a reactive saccade condition, in which subjects directed their gaze immediately after the lateral dot was presented. A vertical bar stimulus was flashed synchronized with the saccade execution inside its trajectory (with a probability of 80% across all trials) and subjects had to report whether they saw the bar. A model was adjusted considering the nature of the trial (voluntary or reactive) and the luminance of the bar to predict response correctness. Reactive saccades were found to have slightly increased peak velocity. Detection thresholds were higher for voluntary saccades, suggesting a stronger suppression effect for voluntary rather than reactive saccades.

A dot flashed twice at the same position is perceived as moving in a direction opposite to the direction of a moving target during smooth pursuit eye movements, a process named movement-induction apparent motion (MIAM) (Gosselin and Faghel-Soubeyrand, 2017). This process is similar to apparent motion (AM), in which two neighboring flashing lights are perceived as a unique light moving, during eye fixations. Six adult participants participated in the task that demonstrated this effect (Gosselin and Faghel-Soubeyrand, 2017). An EyeLink II system by SR Research sampling at 250 Hz was used for eye recordings, with participants at 66 cm using a chin rest to stabilize the head. Stimuli were presented on a computer display with 1,920 × 1,080 pixel resolution. Subjects had to follow a moving red do with the eye across the horizontal axis in a display; when the object reached the center of the monitor, two flashing white disks were presented consecutively. The horizontal displacement of the second position relative to the first was varied, to find the point at which the perceived motion was not reported reliably.

In the studies reported in this section, participants were under explicit instructions to execute saccades, either to the target stimuli they had to detect or to other auxiliary stimuli (so that the target stimuli could be presented while the eye movement was being executed). Conventional button presses techniques were used in those studies, and the eye tracking technique played the role of determining the conditions under which the response was given.

## 6. Using eye tracking to map the visual field

### 6.1. Using statistics of saccades and fixations during visual search

Both contrast and spatial frequency perception vary with increasing eccentricity from the fovea (Loschky and McConkie, 2002). Changes in the visual field periphery, either artificially via stimulus manipulation, or due to clinical conditions, might impact the participant’s visual search strategy in a quantifiable way. In the studies described in this section, participants were under free viewing conditions of naturalistic scenes, and the statistics of saccade and fixation frequency is the variable of interest. We present an example of a foveated rendering study (Loschky and McConkie, 2002), which investigated the degree of real-time spatial filtering on visual search strategies, and a study that investigated the effects of a clinical condition (Smith et al., 2012) on visual search.

In the study by Loschky and McConkie (2002) 15 adult participants with normal vision were tasked with observing 2 sets of 15 monochrome photographs, which were blurred in real-time, matching the decreasing spatial frequency threshold as eccentricity increases from the fovea. A custom-built system sampling at 1,000 Hz was used. The authors presented the images in 9 different conditions (using 1, 4, or 7 wavelet sub-bands crossed with three filtering window radii of 1.6, 2.9, and 4.1 radii). In the first visual search task, participants were instructed to find a specific small object in the scene and press a button when they did. In a second visual memory task, participants were presented with a series of five images, and then after some time presented with matched five images, three of which were the same as the ones before, and two of which were exactly modified, and participants had to make a 2AFC to determine whether the images were the same or modified. As the radius of the high-resolution window decreased, search times increased. At the last 4.1 radii, search times were no different than the condition without any filtering. But the authors did not find any effects of the level of filtering resolution or its interaction with the window radius. For the memory task, window size did not impact the mean number of fixation, fixation duration, or saccade length. But the peripheral filter level impacted saccade length, with increasing saccade lengths for increased filtering level.

Smith et al. (2012) investigated the performance of glaucoma patients in a visual search task. Forty patients with binocular glaucomatous visual field deficits and the same number of control participants had their performance on a visual search task evaluated (where they had to look for an object in a series of photographs). Stimuli were presented on a 1,600 × 1,200 px display. An EyeLink II system sampling at 500 Hz was used. The average number of saccades per second, average saccade amplitude and average search duration across trials were the variables of interest. Patients were found to perform 5.6% fewer saccades on average, although the saccade amplitudes did not differ significantly between groups. The authors identified a modest relationship between poor contrast sensitivity (rho = 0.42) and severe visual field defects (rho = 0.34) and smaller rates of eye movements. In the patient group, average detection time was related to a smaller rate of saccades (*r* = −0.65), a relationship not observed in the patient group.

### 6.2. Using eye tracking to monitor fixation

Eye tracking allows determination of the eccentricity of a stimulus without constraining participant movement: it can be used either as a tool to ensure the participant fixates at a point while test stimuli are presented peripherally, or can be integrated into stimulus presentation software to adaptively present stimuli at a desired eccentricity and polar angle depending on the current fixation point. In both conditions, traditional button press responses or saccades after stimulus onset have been used as dependent measures. The current section will review studies that employed the first strategy (monitoring of fixation), while the next section will review studies that employed the second strategy (adaptively changing stimulus position).

Himmelberg et al. (2020) presented Gabor patterns of varying spatial frequency and size at different eccentricities and meridians in the visual field relative to a central fixation point and participants were asked to solve a 2AFC task to inform the stimulus orientation. Nine adult observers with normal vision participated in the task. Stimuli were presented on a 21-inch display. An EyeLink 1000 system sampling at 1,000 Hz was used for the task. Participants sat at 57 cm and had their head stabilized with a chin rest. The authors identified that contrast sensitivity decreased as a function of eccentricity. This effect of stimulus eccentricity may be canceled by increasing the stimulus size, which counters the effect of a smaller cortical representation for peripheral stimuli. Contrast sensitivity was higher in the horizontal meridians compared to the lower vertical meridian, which in turn was height than the upper vertical meridian (horizontal and vertical visual asymmetries).

Changes in visual acuity dependent on the eccentricity and polar angle (24 positions) in the visual field were investigated by Barbot et al. (2021). Observers, which were 14 adults with normal or corrected-to-normal vision, had to perform a 2AFC to determine the orientation of grating stimuli varying in spatial frequency, and acuity was estimated as the 75% hit rate. Stimuli were presented on a 1,600 × 1,200 px CRT monitor, with patients at a distance of 57 cm and their heads stabilized in a chin rest. An EyeLink 1000 eye-tracking system was used for gaze recording. The authors identified no difference between the left and right hemifields. There were, however, significant asymmetries when comparing the horizontal with the vertical meridian, and when comparing the upper with the lower meridian (favoring the lower meridian), a result similar to previous studies. The asymmetries were more pronounced in the cardinal directions and decreased with the polar angle away from the cardinal directions.

Visual field perimetry was studied by Vullings and Verghese (2021) in a flashing dot detection task presented to the subjects at different locations in a grid arrangement while their fixation was monitored. The authors examined nine individuals with macular degeneration and four age-matched controls. Stimuli were presented on a 40.36° × 30.27° projection screen at a viewing distance of 1 m An EyeLink system sampling at 1,000 Hz was used for gaze recording. The visual field losses identified by relating the position of the dot with the proportion of correct responses were found to match the position and extension of scotomata identified in the optical coherence tomography microperimetry examination [scanning laser ophthalmoscope/optical coherence tomography (SLO/OCT)]. While the SLO/OCT technique only allows a monocular visual field mapping, the authors pointed out that eye tracking technique allows the mapping of both a monocular and a binocular visual field.

### 6.3. Adaptively choosing stimulus position for perimetry

One of the motivations for the development of novel perimetry techniques based on eye tracking is the examination of preschooler participants. Traditional visual field examination with this population is challenging (Murray et al., 2018): in the clinical confrontation test, one examiner holds the child’s fixation and watches gaze changes while a second examiner introduces stimuli from behind the child from the sides of his visual field. The technique is qualitative and does not give precise quantitative information about the visual field. Standard automated perimetry, in turn, might be impractical, since children might not understand or comply with verbal instructions (Murray et al., 2018).

Murray et al. (2018) developed a perimetry solution called saccadic vector optokinetic perimetry (SVOP), and tested its application in brain tumor patients. The test does not require the child to maintain fixation on a static fixation target for long intervals or respond with a button press (as is typical of traditional perimetry), but instead, it relies on natural saccade and fixation behavior. Sixteen patients between 2.9 and 15 years with a clinical diagnosis of brain tumors were referred for evaluation, of which 12 could be tested successfully. Stimuli (bright dots subtending 0.43° of visual angle presented against a darker background) were presented on a 20-inch LCD display, and the Tobii systems X50 and IS-1 were used in the examinations. A ludic 5-point calibration procedure taking between 20–30 s was used. Stimuli were presented at varying eccentricities and polar angles in the visual field, while the fixation point was positioned on the same display coordinate as the last test stimulus. The test had a sensitivity of 100% and a specificity of 50% for detecting visual field deficits. The test dynamically adjusts stimulus position based on the current point of gaze, and therefore does not require the patient to comply with fixation instructions or be physically restrained (which are required in traditional infant perimetry assessments). For the group of children that could also be tested with the traditional Goldmann perimetry test, there was good agreement with the results of both tests. Perperidis et al. (2021) later tested 13 younger patients with normal vision and without any neurological issues using the same procedure, with ages between 3.5 and 12.0 months of age. The exam proceeded with an increasingly dense sampling of the whole visual field. Only infants that completed tests with fewer testing points advanced to later stages. Twelve out of thirteen infants could complete a 4-point screening test; 7 completed the 12-point test; 4 completed the 20-point test; and 3 completed the 40-point test.

Jones (2020) describe a procedure named “Eyecatcher,” an eye tracking perimetry test adequate for use with children and neurological patients. The designed task is similar to the author’s previous study (Jones et al., 2014) (that tested visual acuity in infants) with respect to stimulus positioning, which was adaptively chosen based on the current fixation point. Adults with normal or corrected-to-normal vision participated in the novel proposed procedure and in the Humphrey Field Analyzer (HFA) exam. White dots (0.43° with varying luminance arranged in a 24-2 perimetric grid) similar to the Goldman stimuli used in standard perimetry testing were presented on a 59.7 × 33.6 LCD computer display using a homogeneous 10 cd/m2 background while participant’s eyes were monitored without head restriction or requirement for maintaining fixation (stimuli were presented relative to the current fixation point). A Tobii EyeX system sampling at 50 Hz was used for recording. Participants were instructed to simply look at the display, and responses were saccades naturally occurring toward the stimuli. For each stimulus position, differential light sensitivity was calculated (smallest difference between the dot and the background), and thresholds were calculated as the differential light sensitivity (DLS) where 50% of stimuli could be detected by saccades. The paired scores between the two techniques were not significantly different (*t* = −0.86, *p* = 0.39), and scores were significantly correlated (*r* = 0.59, *p* < 0.001). Visual sensitivity maps were similar to the traditional technique, with the peak near the fovea and a highly salient blind spot.

## 7. Discussion

### 7.1. Limitations

Since our article cannot be characterized as a systematic review, it might have omitted a few works that qualified for the proposed inclusion criteria. The aim of the review was not to be comprehensive, but rather to be reasonably representative of the methodological diversity found in contemporary psychophysics literature. To ensure representativeness, we included studies with very different research objectives (basic perceptual processes and clinical validation studies) and using diverse solutions for gaze recording and stimulus presentation. While we did not adopt time of publication as a strict inclusion criteria, our work focused on research literature over the last two decades, mostly reflecting the increased popularity of video-based eye tracking over this period (Carter and Luke, 2020). Therefore, another limitation of our study is that it might have omitted older works that qualify for the current discussion, due to space constraints.

Our review also presented a short summary of each study’s findings, in most cases missing the required level of detail for a more thorough critical evaluation of the study’s impact in its research area. Results were presented only in their immediate relation to the present methodological discussion, and the presentation might have missed aspects that would otherwise be included if the review focus were more directed to one of the many (and rather diverse) ranges of research questions treated. Our work also focused on how participant’s gaze responses relate to psychophysical variables of interest, so other relevant technical aspects that researchers should keep in mind when developing psychophysical tasks (such as distance-to-monitor, screen refresh rate, color gamut and stimulus synchronization) were not discussed.

With those limitations in mind, we discuss a few common points in the studies reviewed that might be useful to inform future task design.

### 7.2. Can eye movement responses be an objective index of stimulus detection in psychophysical tasks?

A few of the studies reviewed here propose eye tracking as an objective index of stimulus detection, in the sense of removing the need for an external observer to assess participant behavior as in preferential looking studies (Sturm et al., 2011; Hathibelagal et al., 2015; Zhuang et al., 2021; Esteban-Ibañez et al., 2022), or in the sense of eliciting responses that are not under participant voluntary control such as the OKN (Hyon et al., 2010; Schwob and Palmowski-Wolfe, 2019; Essig et al., 2021), microsaccades (Scholes et al., 2015; Denniss et al., 2018), or smooth pursuit (Mooney et al., 2018).

The preferential looking studies reviewed here use as their dependent measure the relative fixation time spent on the target stimulus, which is compared either to the fixation time outside the stimulus region (display background) or to the fixation time on other candidate stimulus (Sturm et al., 2011; Chang and Borchert, 2021; Vrabič et al., 2021). While eliminating the need for an external observer to make qualitative judgments about the participant’s gaze, each study defines its own criteria for stimulus detection, which has been shown to impact measured thresholds (Vrabič et al., 2021). The advantage of the eye tracking technique is that the judgment of the observer is replaced by an automated and in principle reproducible detection algorithm, that can be applied uniformly to all participants within a study. The existing variability in criteria across studies makes comparing results of different studies challenging, since those criteria might be based on unpublished pilot studies and/or formal mathematical analysis of task-specific characteristics (Jones et al., 2014). Ideally, studies should publish a detailed analysis off how detection criteria are chosen, and demonstrate how sensitive the final estimates are to different and equally reasonable criteria for detection.

Studies employing the statistics of eye movements in visual search (Loschky and McConkie, 2002; Smith et al., 2012) are similar to preferential looking studies in that participants perform the task according to fairly generic instructions, but differ in that there is not a specific fixation target, and therefore there is no need to determine particular detection criteria. Eye movement statistics (such as the number of saccades and mean saccade amplitude) are then averaged across trials and compared across different experimental conditions. While those studies cannot be used to define psychophysical thresholds (therefore have limited clinical applicability) they might inform aspects of the design of studies that are used to determine thresholds, such as participant-specific baseline rates of eye movements.

When eye movement recordings are qualified as objective, usually one of the following meanings are implied: (1) responses cannot possibly be inhibited by the participants (as in OKN tasks) or (2) the response happens with reasonably high probability in the user natural behavior (as in reactive saccade tasks). Although smooth pursuit movements cannot be started voluntarily by participants in the absence of a moving stimulus (therefore being a good indicator of stimulus detection at their onset), the movement can be inhibited once started (Missal and Heinen, 2017). OKN cannot be voluntarily suppressed except by the superposition of a fixed object or percept on top of the fixation point in the OKN-inducing stimulus (Howard et al., 1989), a fact that was explored by a few studies to determine spatial frequency thresholds (Hyon et al., 2010; Schwob and Palmowski-Wolfe, 2019). Although both pursuit and saccades are objective in the sense of not depending on subject initiation or spontaneous visual exploration, they still assume a collaborative subject to maintain fixation throughout the task duration.

Some studies also propose that reactive saccades might be treated as an objective measure of stimulus detection (Murray et al., 2018; Essig et al., 2022). Reactive saccades are very reasonable indices of stimulus detection (hit trials) when one considers (1) the close temporal association between stimulus and eye movement, since reactive saccades tend to have lower latencies than voluntary saccades (Walker et al., 2000) and (2) the baseline probability of fixating on the stimulus region (irrespective of detection) is low, given a sufficiently small stimulus area relative to the display size (Jones et al., 2015). Saccades are under voluntary control, and although the most natural tendency is to fixate on suddenly appearing peripheral stimuli, even under fairly generic verbal instructions such as “look toward the screen” (Jones, 2020), less cooperative subjects might neglect to look at the stimuli. Reactive saccades toward peripheral stimuli can be inhibited, save for a short period after saccade planes are formulated (Kornylo et al., 2003). The question is less straightforward when one needs to consider whether the failure to produce a saccade means non-detection (miss trials). A more thorough analysis of the lapse rate of reactive saccades near the stimulus threshold (assuming a collaborative participant) is an open question that merits further exploration: can the failure to produce a saccade toward supra-threshold stimulus be due to the stimulus being insufficiently salient to elicit this response? Can saccades near stimulus threshold be a product of voluntary (instead of reflex) eye movements?

The type of movement used by the researcher is not the sole factor that should be used in establishing whether the nature of the task is subjective or objective. The analysis must also consider the verbal instructions given, whether they were given at all (as is the case for preverbal infants), and what data quality steps were made (i.e., were saccades removed or ignored in a study focusing on smooth pursuits? Were trials without saccades in the instructed direction removed or kept?). The presence of verbal instructions is a fundamental methodological aspect because they set a goal. When participants are actively engaged in some activity, scene aspects relevant to the current goal are much better predictors of fixation than lower-level visual features (Schütz et al., 2011).

### 7.3. How do eye movement responses compare with traditional tasks requiring manual or verbal responses?

While studies interested in higher-level cognitive processes and abilities (attention, memory) might present only stimuli above participant sensory thresholds, in psychophysical studies participant performance must be characterized both when stimuli are detected (hit trials) as when they are not (miss trials). The operationalization of detection performance in psychophysical tasks with responses in the spatial dimension afforded by the eye tracker, however, is not immediately clear. Different detection criteria might lead to different threshold estimates. Defining what a “hit” trial is in terms of relative fixation time within a target stimulus (dwell time) or a saccade latency with respect to stimulus onsets does not imply automatically that “miss” trials are simply trials that do not fulfill those criteria, since the researcher still needs to decide which trials to exclude from further analysis based on considerations such as inattentiveness to the task, excessive blinking or participant movement, or non-compliance with task demands. Repeating trials if the participant was not fixating on a desired area or was blinking (Essig et al., 2022), or removing recording intervals when the participant’s eye was closed (Ming et al., 2016) might have a direct effect on results. The possibility that repeated or removed trials are related to the variable of interest (e.g., participants might look away from the trackable area more frequently on trials where the stimulus is non-detected) cannot be discarded.

A basic step in psychophysical task design is to define a psychometric function that relates some physical stimulus property of interest such as spatial frequency or contrast to some characteristic of participant response. Traditionally, the proportion of correct responses (hit trials) is one of the main response characteristics taken as the dependent variable of interest. In many of the reviewed studies, this variable was still used by reducing the spatiotemporal eye tracking measures to a binary variable. The analysis is straightforward when reflexive responses such as the OKN are used: this maps directly to a binary variable after the gaze feature extraction stage (OKN present/absent) (Hyon et al., 2010). In a reactive saccade paradigm, the single target of the first saccade after stimulus onset might be used instead (Essig et al., 2022). In preferential looking studies, detections are determined when most of the trial was spent fixating a region of interest around a stimulus, with 50–60% adopted as common criteria for “most” (Hathibelagal et al., 2015; Chang and Borchert, 2021; Vrabič et al., 2021). A less conventional analysis, performed by Sturm et al. (2011), used relative fixation time directly as the dependent variable of interest in the psychometric function.

The quantification of sensitivity indices from the measured psychometric functions was one of the major breakthroughs of psychophysics, which allowed the formal treatment of effects unrelated to the detection task itself (such as accommodating more conservative or liberal observers, and treating inattention and fatigue as noise) (Stanislaw and Todorov, 1999; Geisler, 2011). When the eye tracer is used just as a tool to remove trials where participants did not maintain fixation on an expected region, or to guarantee a response is given within a specific time frame (near eye movement or stimulus onset), the sensitivity analysis does not diverge significantly from traditional psychophysical technique, since manual responses are still the variable of interest. This is a different matter altogether if the recorded spatial dimension (gaze position) is taken as the response medium. In studies that used regions of interest around the target stimulus, the spatial distribution of fixation targets has been modeled as a continuous two-dimensional uniform distribution (Jones et al., 2014; Jones, 2020), or as a discrete uniform distribution when the total stimuli area covers the whole screen (Sturm et al., 2011), with individual detection criteria being formulated in terms of a likelihood-ratio criteria, with the alternative model distribution based either on empirical estimates from many subjects (Sturm et al., 2011) or by approximation, as a Gaussian distribution peaked at stimulus position (Jones, 2020). In general, the baseline probability of false positives in detection tasks will be proportional to effective display size and inversely proportional to the stimulus size, so the spatial arrangement of stimuli and their relative size will have an impact on sensitivity analysis (Jones et al., 2015).

There is no guarantee that any detection criterion based on the proportion of time fixating on certain regions might apply equally well to all participants. One way of reducing participant variability is to derive individual baseline eye movement characteristics. Denniss et al. (2018), for example, compared the microsaccade rate during stimulus presentation to the baseline rate of each participant and the maximum rate for each participant, that is, the rate at maximum stimulus contrast (Denniss et al., 2018). The microsaccade rate was then modeled as two alternative binomial models, with the proportion parameter set at the baseline rate or at the rate for the maximum contrast stimulus, and the comparison of the models via the log-likelihood ratio criteria was done. Analysis of this nature might be extended to other task paradigms, as a way to accommodate different visual search strategies individual participants might have, for example.

## 8. Conclusion

Eye tracking technique offers new opportunities for psychophysical research in terms of expanding testable populations and psychological processes, but it also brings new challenges. While manual responses in a forced choice or detection task map unambiguously to a discrete categorical variable, eye tracking research requires a series of decisions from the researcher on how to process the gaze data. Eye movement features must not only have a precise mathematical and/or statistical definition to be separable in the raw gaze signal, but must also be related to the subject detection or discrimination performance in an objective and reproducible way. While the feature extraction steps have been extensively developed and might already be implemented in current eye tracking systems, it is still up to the researcher to determine how this output maps to the variable of interest. The evaluation of clinical populations without reliance on verbal instructions is one of the main motivations for eye tracking measurements. In such situations, where participants are minimally constrained in how they should respond, the challenge is even greater, and extra care must be applied in defining a psychometric function and during sensitivity analysis to produce a valid mapping between eye movement and stimulus detection. We hope that our survey of the current literature in psychophysical eye tracking research might help with the clarification and categorization of the different strategies that have been employed with regard to this second step.

Apart from being an alternative for examining clinical populations, eye tracking can have a complementary role in tasks requiring voluntary patient responses. The technique can also greatly improve the accuracy (fixation points are known rather than assumed) and ergonomics (minimizing physical restraint requirements) of visual field evaluations, which typically require patient collaboration to hold fixation while physically constrained. As the challenges associated with this research area are solved, we expect research tasks might translate into novel diagnostic tools at the disposal of eye care professionals (Mooney et al., 2018; Chang and Borchert, 2021), as has happened with many other classical psychophysical tasks (Fitzke, 1988).

## Author contributions

DS: literature review and manuscript writing. DV: manuscript writing and review. Both authors contributed to the article and approved the submitted version.
