# Supplementary material for: A review of experimental task design in psychophysical eye tracking research
Source: Front Hum Neurosci. 2023 Aug 17;17:1112769. doi: 10.3389/fnhum.2023.1112769 (PMC10469886; doi:10.3389/fnhum.2023.1112769)
Supplement: Supplementary file 1 [file Table_1.pdf]

Supplementary Table 1. Summary of the studies included in the current review. Studies are grouped by the main research area according to the categorization proposed at Section 2.

Research area	Study	Experimental paradigm
Stimulus detection and threshold estimation	Kilpelainen et al. (2013)	Reactive saccade
	Zhuang et al. (2021)	Reactive saccade
	Essig et al. (2022)	Reactive saccade
	Sturm et al. (2011)	Preferential looking (fixation time)
	Jones et al. (2014)	Preferential looking (fixation time)
	Hathibelagal et al. (2015)	Preferential looking (fixation time)
	Vrabic et al. (2021)	Preferential looking (fixation time)
	Chang et al. (2021)	Preferential looking (fixation time)
	Esteban-Ibanez et al. (2022)	Smooth pursuit tracking
	Ming et al. (2016)	Smooth pursuit tracking
	Mooney et al., 2018	Smooth pursuit tracking
	Mooney, Alam, Hill, & Prusky, 2020	Smooth pursuit tracking
	Dakin & Turnbul (2016)	Optokynetic nystagmus detection
	Schwob et al. (2019)	Optokynetic nystagmus detection
	Essig et al. (2021)	Optokynetic nystagmus detection
Effects of stimulus properties on fixational eye movements	Bonneh, Adini, & Polat, 2015	Microsaccade measurement
	Scholes et al. (2015)	Microsaccade measurement
	Denniss et al. (2018)	Microsaccade measurement
The effects of eye movements on perception	Schutz et al. (2007)	Smooth pursuit tracking
	Castet et al. (2000)	Voluntary saccade
	Gremmler et al. (2017)	Voluntary saccade
	Gosselin & Faghel-Soubeyrand	Smooth pursuit tracking
Visual field assessment	Loschky et al. (2002)	Statistics of visual search
	Smith et al. (2012)	Statistics of visual search
	Himmelberg et al. (2020)	Fixation monitoring
	Barbot et al. (2021)	Fixation monitoring
	Vullings et al. (2021)	Fixation monitoring
	Murray et al. (2018)	Reactive saccades
	Jones et al. (Jones, 2020)	Reactive saccades
